# Supplementary material for: The Beneficial Effect of Mitochondrial Transfer Therapy in 5XFAD Mice via Liver–Serum–Brain Response
Source: Cells. 2023 Mar 24;12(7):1006. doi: 10.3390/cells12071006 (PMC10093713; doi:10.3390/cells12071006)
Supplement: Supplementary file 1 [file cells-12-01006-s001.zip › Supplemental Tables, Figure, Methods 14.2.23A.pdf]

Table S1. **Altered proteins due to the mitochondrial treatment showing "Treatment Rescued" or "Treatment effect" in hippocampus (total homogenates and aggregated fraction) and in liver.**

**AD vs AD-Treated - hippocampus homogenates**				
**Protein names**	**Gene names**	**p-value**	**Fold Change**	**Effect**
AD>AD-Treated				
UPF0461 protein C5orf24 homolog	MGI:1925771	0.000*	39.31	Treatment Rescued
Cyclin-dependent kinase 9	Cdk9	0.020*	29.60	Treatment Rescued
E3 ubiquitin-protein ligase MIB2	Mib2	0.012*	24.25	Treatment Effect
Kelch-like ECH-associated protein 1	Keap1	0.007*	19.17	Treatment Effect
Gamma-aminobutyric acid receptor subunit alpha-4	Gabra4	0.003*	19.10	Treatment Effect
Riboflavin kinase	Rfk	0.039*	16.25	Treatment Effect
Gamma-aminobutyric acid receptor subunit alpha-3	Gabra3	0.051^	14.65	Treatment Effect
Myocardin-related transcription factor A	Mrtfa	0.011*	13.04	Treatment Effect
Gigaxonin	Gan	0.011*	12.32	Treatment Effect
Ras suppressor protein 1	Rsu1	0.048*	12.16	Treatment Rescued
E3 ubiquitin-protein ligase RNF34;E3 ubiquitin-protein ligase MIB1	Mib1	0.008*	9.24	Treatment Effect
Cell division cycle protein 23 homolog	Cdc23	0.124^	8.82	Treatment Effect
Serine/arginine-rich splicing factor 9	Srsf9	0.064^	8.36	Treatment Effect
Squamous cell carcinoma antigen recognized by T-cells 3	Sart3	0.130^	7.92	Treatment Effect
Nuclear receptor-interacting protein 3	Nrip3	0.036*	7.05	Treatment Rescued
Potassium voltage-gated channel subfamily B member 1	Kcnb1	0.065^	6.5	Treatment Effect
Amyloid-beta 1-42 protein	APP 1-42	0.058^	5.73	Treatment Rescued
MI domain-containing protein	Gm13695	0.187^	5.05	Treatment Effect
Frataxin, mitochondrial	Fxn	0.094^	4.66	Treatment Effect
Chloride intracellular channel protein 1	Clic1	0.001*	3.57	Treatment Rescued
Nuclear cap-binding protein subunit 2	Ncbp2	0.198^	3.15	Treatment Effect
V-type proton ATPase 16 kDa proteolipid subunit	Atp6v0c	0.004*	2.53	Treatment Effect
Rab effector MyRIP	Myrip	0.003*	2.47	Treatment Effect
GRB10-interacting GYF protein 1	Gigyf1	0.024*	2.37	Treatment Rescued
Amyloid-beta 1-40 protein	APP 1-40	0.052^	2.26	Treatment Rescued
Splicing regulatory glutamine/lysine-rich protein 1	Srek1	0.002*	2.05	Treatment Effect
Arginine and glutamate-rich protein 1	Arglu1	0.003*	1.91	Treatment Effect
AD<AD-Treated				
Vitamin K-dependent protein S	Pros1	0.010*	0.01	Treatment Rescued
RING finger protein 141	Rnf141	0.011*	0.03	Treatment Rescued
Regulator of nonsense transcripts 2	Upf2	0.010*	0.04	Treatment Effect
Apolipoprotein D	Apod	0.004*	0.05	Treatment Rescued
Mediator of RNA polymerase II transcription subunit 1	Med1	0.010*	0.06	Treatment Rescued
Betaine--homocysteine S-methyltransferase 1	Bhmt	0.007*	0.07	Treatment Rescued
Protein PBDC1	Pbdc1	0.067^	0.08	Treatment Rescued
39S ribosomal protein L38, mitochondrial	Mrpl38	0.013*	0.08	Treatment Rescued
Nuclear factor 1 A-type	Nfia	0.011*	0.09	Treatment Rescued
Bromodomain-containing protein 2	Brd2	0.001*	0.09	Treatment Rescued
Serine/threonine-protein phosphatase 4 catalytic subunit	Ppp4c	0.013	0.11	Treatment Rescued

28S ribosomal protein S6, mitochondrial	Mrps6	0.071^	0.12	Treatment Rescued
Noelin-2	Olfm2	0.037*	0.13	Treatment Effect
5-nucleotidase	Nt5e	0.109^	0.13	Treatment Rescued
Fibrinogen beta chain	Fgb	0.069^	0.14	Treatment Rescued
Putative RNA-binding protein Luc7-like 1	Luc7l	0.044*	0.16	Treatment Effect
Band 3 anion transport protein	Slc4a1	0.180^	0.23	Treatment Rescued
Protein XRP2	Rp2	0.021*	0.40	Treatment Effect
Immunoglobulin kappa constant	Igkc	0.018*	0.43	Treatment Rescued
Carboxypeptidase M	Cpm	0.028*	0.55	Treatment Effect
Nesprin-2	Syne2	0.001*	0.55	Treatment Effect

AD vs AD-Treated - hippocampus aggregates

Protein names	Gene names	p-value	Fold Change	Effect
AD>AD-Treated				
E3 ubiquitin-protein ligase MIB2	Mib2	0.012*	21.9	Treatment Effect
E3 ubiquitin-protein ligase Praja-1	Pja1	0.009*	17.72	Treatment Effect
Serine hydroxymethyltransferase, mitochondrial	Shmt2	0.006*	17.37	Treatment Effect
UPF0160 protein MYG1 (exonuclease), mitochondrial	Myg1	0.013*	15.16	Treatment Effect
Myosin-4	Myh4	0.124^	14.34	Treatment Rescued
Trafficking protein particle complex subunit 5	Trappc5	0.040*	13.32	Treatment Rescued
Gamma-aminobutyric acid receptor subunit alpha-5	Gabra5	0.053^	13.32	Treatment Effect
Ras-related protein Rab-30	Rab30	0.085^	10.18	Treatment Effect
Electron transfer flavoprotein-ubiquinone oxidoreductase, mitochondrial	Etfdh	0.070^	9.62	Treatment Effect
E3 ubiquitin-protein ligase RNF34	Rnf34	0.008*	8.92	Treatment Effect
Ras-related protein Rab-2B	Rab2b	0.080^	8.92	Treatment Effect
ADP-ribosylation factor GTPase-activating protein 1	Arfgap1	0.137^	8.72	Treatment Effect
7-methylguanosine phosphate-specific 5-nucleotidase	Nt5c3b	0.161^	5.55	Treatment Rescued
HUMAN Amyloid-beta 1-42 protein	APP1-42	0.060^	5.54	Treatment Rescued
Surfeit locus protein 1	Surf1	0.141^	5.2	Treatment Effect
Small nuclear ribonucleoprotein Sm D1	Snrpd1	0.170^	4.88	Treatment Rescued
Eukaryotic initiation factor 4A-II	Eif4a2	0.184^	4.44	Treatment Effect
Zinc transporter 1	Slc30a1	0.142^	4.38	Treatment Effect
Coatomer subunit zeta-1	Copz1	0.117^	3.36	Treatment Rescued
HUMAN Amyloid-beta 1-40 protein	APP1-40	0.048*	2.39	Treatment Rescued
Metallothionein-3	Mt3	0.092^	2.1	Treatment Effect
ATP-binding cassette sub-family E member 1	Abce1	0.077*	1.89	Treatment Effect
Eukaryotic translation initiation factor 3 subunit H	Eif3h	0.013*	1.88	Treatment Effect

Cytochrome c oxidase subunit 6A1, mitochondrial	Cox6a1	0.155^	1.87	Treatment Effect
Eukaryotic translation initiation factor 4H	Eif4h	0.080^	1.79	Treatment Effect
Proteasome subunit alpha type-3	Psma3	0.037*	1.79	Treatment Rescued
Metallothionein-1	Mt1	0.168^	1.75	Treatment Effect
<u>AD<AD-Treated</u>				
Glutathione hydrolase 7	Ggt7	0.010*	0.05	Treatment Rescued
Cytochrome c oxidase assembly factor 3 homolog, mitochondrial	Coa3	0.067^	0.07	Treatment Rescued
Sodium-dependent serotonin transporter	Slc6a4	0.0137*	0.13	Treatment Effect
Band 3 anion transport protein	Slc4a1	0.074^	0.16	Treatment Rescued
E3 ubiquitin-protein ligase MARCHF5	Marchf5	0.082^	0.24	Treatment Effect
Mitochondrial dynamics protein MID51, (mitochondrial elongation factor 1)	Mief1	0.193^	0.25	Treatment Effect
Prostaglandin reductase 2	Ptgr2	0.162^	0.48	Treatment Rescued
NADH dehydrogenase [ubiquinone] 1 alpha subcomplex subunit 11, mitochondrial	Ndufa11	0.0143*	0.52	Treatment Effect

AD vs AD-Treated - liver homogenates

**Protein names**	**Gene names**	**p-value**	**Fold Change**	**Effect**
<u>AD>AD-Treated</u>				
R3H and coiled-coil domain-containing protein 1	R3hcc1	0.011*	594.07	Treatment Rescued
Carnitine O-palmitoyltransferase 1, muscle isoform	Cpt1b	0.176^	38.31343	Treatment Effect
Nucleolar protein 16	Nop16	0.01*	33.48	Treatment Effect
Laminin subunit beta-1	Lamb1	0.118^	28.98864	Treatment Effect
Insulin-like growth factor-binding protein 2	Igfbp2	0.011*	17.03	Treatment Effect
Phosphatidylinositol 3-kinase regulatory subunit alpha	Pik3r1	0.059^	13.95	Treatment Rescued
Heat shock protein beta-1	Hspb1	0.139^	12.43845	Treatment Effect
Pancreatic alpha-amylase;Aamy domain-containing protein	Amy2	0.191^	11.67671	Treatment Effect
Perilipin-1	Plin1	0.09^	8.007248	Treatment Effect
Phosphoenolpyruvate carboxykinase [GTP], mitochondrial	Pck2	0.089^	6.23	Treatment Effect
Phosphatidylcholine-sterol acyltransferase	Lcat	0.184^	6.010514	Treatment Rescued
Deubiquitinase OTUD6B	Otud6b	0.195^	5.64	Treatment Rescued
Serine/threonine-protein phosphatase 4 regulatory subunit 3A	Ppp4r3a	0.181^	5.158669	Treatment Rescued
Serine protease inhibitor A3M	Serpina3m	0.148^	1.82	Treatment Rescued
Beta-galactosidase	Glb1	0.042*	1.74	Treatment Effect
<u>AD<AD-Treated</u>				
Glutathione S-transferase pi 3	Gstp3	0.009*	0.03	Treatment Rescued
Caveolin-1	Cav1	0.162^	0.074116	Treatment Rescued
Autophagy protein 5	Atg5	0.075^	0.083115	Treatment Rescued
Target of rapamycin complex subunit LST8	Mlst8	0.068^	0.095417	Treatment Rescued

Protein phosphatase 1 regulatory subunit 21	Ppp1r21	0.115^	0.10	Treatment Rescued
Bis(5-adenosyl)-triphosphatase	Fhit	0.108^	0.106667	Treatment Rescued
AH receptor-interacting protein	Aip	0.084^	0.12	Treatment Effect
Hemoglobin subunit beta-2	Hbb-b2	0.067^	0.158829	Treatment Effect
Nucleobindin-2	Nucb2	0.083^	0.23	Treatment Effect
Protein PML	Pml	0.036*	0.48	Treatment Effect

Presented as AD>AD-Treated (treatment decreased protein level) and AD<AD-treated (treatment increased protein level).

^ "Treatment Rescued": trend of difference in AD vs AD-Treated, and significant or trend in AD vs Non-AD

^ "Treatment Effect": trend of difference in AD vs AD-Treated (mostly with significant or trend AD-Treated vs Non-AD)

<u>Table S2:</u> Altered metabolites in liver, comparing AD vs non-AD, treated AD vs non-AD, and AD vs treated-AD-mice.

Meatabolite	AD (M±SEM)	Non-AD (M±SEM)	P value	AD-treated (M±SEM)	Non-AD (M±SEM)	P value	AD (M±SEM)	AD-treated (M±SEM)	P value	Treatment Effect
**Acylcamitines**										
Hydroxytetradecenoylcarnitine	0.114±0.011	0.094±0.007	0.035	0.089±0.019	0.094±0.007	0.649	0.110±0.009	0.085±0.018	0.073^	Treatment rescued
Hexadecenoylcarnitine	0.107±0.012	0.079±0.0009	0.044	0.065±0.033	0.079±0.001	0.486	0.103±0.017	0.065±0.028	0.076^	Treatment rescued
Hexanoylcarnitine	0.139±0.013	0.138±0.015	0.943	0.109±0.012	0.138±0.015	0.028	0.134±0.013	0.105±0.012	0.021	Treatment effect
**Sphingolipids**										
C24:1-OH Sphingomyelin	0.876±0.132	0.564±0.034	0.004	0.681±0.127	0.564±0.035	0.158	0.844±0.115	0.656±0.124	0.079^	Treatment rescued
SM(d18:0/20:2)	0.404±0.016	0.330±0.034	0.031	0.327±0.075	0.330±0.035	0.955	0.389±0.033	0.316±0.074	0.147^	Treatment rescued
**Amino acids**										
Ala	823.369±167.367	812.010±68.732	0.915	1016.081±86.341	812.383±68.588	0.011	795.512±169.700	978.437±83.713	0.105^	Treatment effect
Asn	43.982±4.835	48.571±12.088	0.560	67.309±6.650	48.600±12.121	0.035	42.400±4.532	64.807±6.340	0.001	Treatment effect
Asp	60.984±10.365	53.625±7.856	0.332	74.998±13.577	53.650±7.851	0.043	58.829±9.415	72.205±12.942	0.170^	Treatment effect
**Biogenic amines**										
Carnosine	0.767±0.007	0.439±0.055	0.000	0.483±0.147	0.439±0.054	0.636	0.739±0.042	0.464±0.139	0.012	Treatment rescued
Putrescine	0.587±0.296	0.554±0.115	0.850	0.772±0.114	0.554±0.114	0.041	0.565±0.261	0.744±0.113	0.265^	Treatment effect

* Statistical significant

^ "Treatment Rescued": trend of difference in AD vs AD-Treated, and significant in AD vs Non-AD

^ "Treatment Effect": trend of difference in AD vs AD-Treated and significant in AD-Treated vs Non-AD

<u>Table S3:</u> Supplemental Table 3: The relative contribution of the highest impact on the difference between the non-AD, AD, AD-treated groups in VIP scores comparison.

Metabolite	Non-AD	AD	AD-treated	Effect
Hexose	++	-	+	Treatment Rescued
Taurine	-	++	+	Treatment Rescued
Glutamine	+	-	++	Treatment Rescued
Glu	+	-	++	Treatment Rescued
Gly	-	+	++	Treatment Rescued (adaptation)
Asn	-	-	+	Treatment Effect
Asp	-	-	+	Treatment Effect
Ala	-	-	+	Treatment Effect

We referred to the metabolites' effect shown in Fig. 6c on a relatively basis (for example, Hexose having the biggest VIP score in the AD vs non-AD comparison: higher level in the non-AD mice than in the AD-mice, and also higher in non-AD than in the AD-treated mice, with the AD-treated being higher than the AD - therefore grading them as: ++, -, + for non-AD, AD, AD-treated, respectively).

<u>Table S4:</u> Altered metabolites in the serum comparing the treated-AD vs the AD-mice.

Metabolite	AD-Treated (M±SEM)	AD (M±SEM)	P value
C24:1-OH Sphingomyelin	0.45±0.28	0.83±0.21	0.014*
Asp	39.31±20.34	15.97±8.77	0.014*
Carnosine	2.13± 1.95	1.35±0.73	0.132^
Putrescine	1.40±0.344	1.13±0.28	0.133^

* Statistical significant

^ Trend

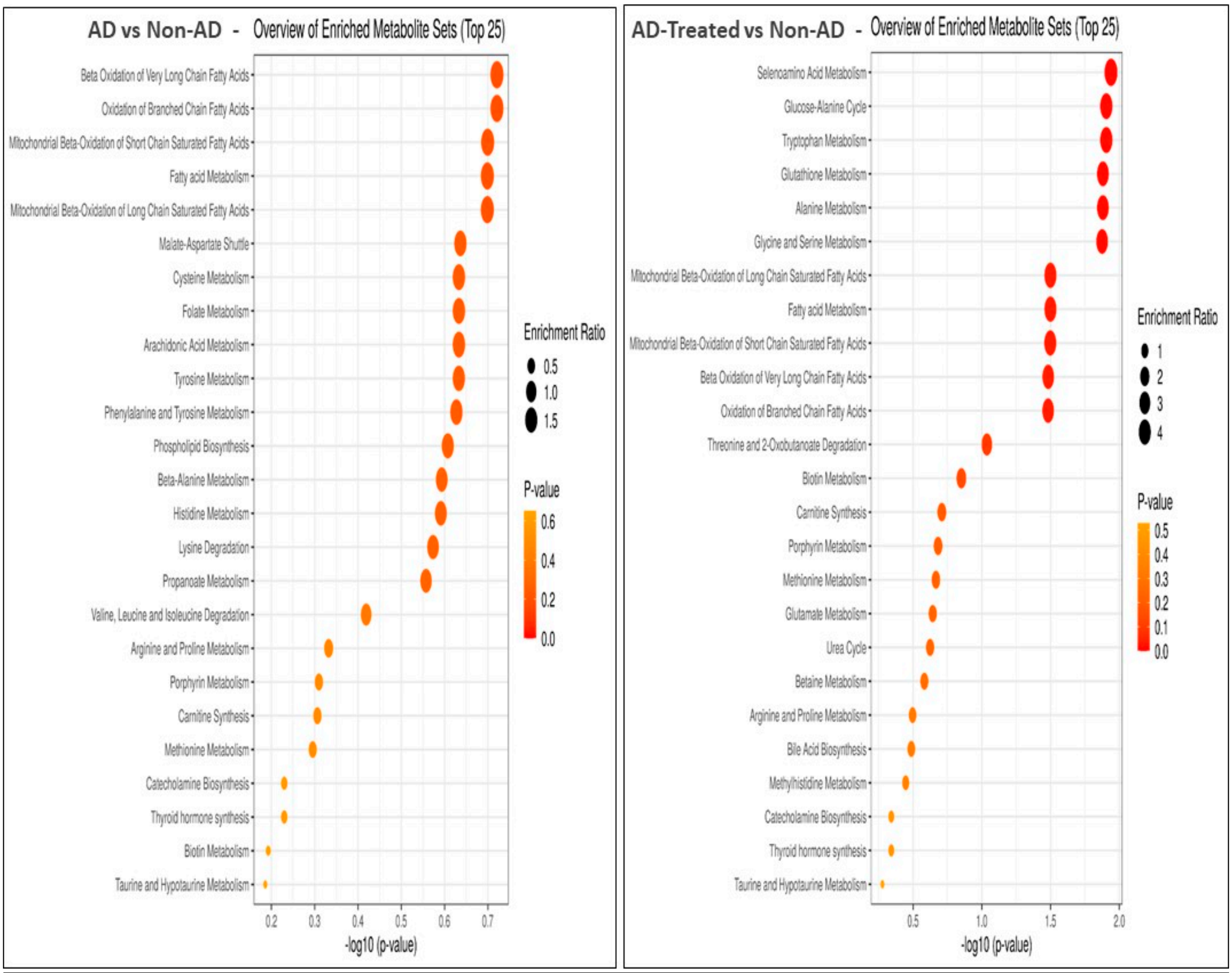

<u>Fig. S1</u>: Metabolomic Pathway Analysis (using the SMPDB pathway libraries as references) for identifying the metabolic pathways enriched by the mitochondria treatment. Presented are comparisons of AD vs non-AD, and AD-Treated vs AD.

<u>Supplemental - Methods</u>

<u>Mitochondria isolation</u>

HeLa DsRed2-mito cells were collected by trypsinization, suspended in PBS, and centrifuged (5 min, 250 g) twice. Mitochondrial isolation procedures were performed at 4 °C or on ice and in the dark (to prevent fluorescence decay). The centrifuged cells were re-suspended in mitochondrial isolation buffer (320 mM

sucrose, 5 mM Tris-HCl, pH 7.4, 2 mM EGTA), and homogenized with a Dounce homogenizer. Nuclei and cell debris were removed by two centrifugations (5 min, 3000 g) and the supernatant was collected. The supernatant was then centrifuged (10 min, 12,000 g) and the mitochondrial pellet was re-suspended in mitochondrial isolation buffer. Mitochondrial concentration was determined by Bradford-assay. All the experiments were performed with freshly isolated mitochondria. We have shown that this isolation protocol keeps the mitochondria intact and viable, inducing beneficial effects in mitochondria-defective-cells [14].

Cognitive tests

Y-maze

This test evaluates short-term memory. The Y-maze is a three-arm maze with all arms at equal angles, 30 cm in length and 5 cm in width with walls 12 cm high. Mice were initially placed in the middle, and the sequence of arm entries were recorded manually for each mouse over a 6-minute period. The triads with all three arms represented (i.e., ABC, CAB, or BCA but not ABB) - were considered 'correct triads' [29].

Open-field habituation

This test evaluates long term non-associative, non-aversive spatial learning by measuring the decrease in the exploratory activity of the animal in a test session carried out 24 h after the first exploration session (delta of 1st session – 2nd session). Animals were exposed to a novel environment by placing them in a 40 cm × 50 cm × 60 cm open field box. The distance walked was measured for a 5 min period (EthoVision). Twenty-four hours later animals were re-exposed to the same environment and the distance they walked was recorded. Significant shorter distance moved in the test session compared to the first session represented intact learning. The higher the delta of distance moved between Day 1 and Day 2 - the better performance it is [29].

Novel object recognition

The novel object recognition test is used to evaluate cognition, especially non-spatial recognition memory. On the training day, the animals were placed in a 25 × 25 cm arena containing two identical objects for 10 minutes and then returned to their home cages. On the testing day, 24 hours later, the mice placed in the same open arena, with one familiar object and one new object, different in shape, color, and texture from the familiar one. Each mouse was allowed to explore the arena for 4 minutes. The ratio of exploration of the novel object and the total exploration of the two objects were calculated and presented in the figure. The test was performed using the Ethovision 10 system, providing fully computerized, blinded and unbiased measurement. Normal animals tent to explore the new object longer than the old one, indicating normally long-term recognition memory [29, 31].

T-maze

The T maze test was use for assessing the spatial long-term memory, measuring exploratory behavior in animals. Shortly, the maze contains 2 arms of 45 cm length and 10 cm width that extended at a right angle from a 57-cm-long alley. The test comprises two trails with an interval of 24 hours. On the first day mice are placed in the start arm of the maze, and allowed to explore it for 8 minutes, while one of the short arms is closed. On the second day, both arms are open, and the animal is allowed to explore all maze parts for 3 minutes. The number of entries to the unfamiliar arm and the time spent there were recorded. Normal healthy animals prefer to visit the unfamiliar arm of the maze on the second day rather than the familiar arm [29, 31].

Histological studies

Sections were stained for amyloid burden with the mouse α-human Aβ (clone α-6E10, Biolegend CA, USA; 1:750) and Thioflavin S (Sigma Aldrich) to assess the plaque burden. Staining to determine the degree of neuronal loss was performed

using the antibody against the neuronal marker (anti-NeuN, Millipore Burlington, MA, USA; clone A60; 1:500), and the Fluoro-Jade C (FJD) (Sigma Aldrich) staining for degenerative neurons. To follow the distribution of the DsRed-labelled mitochondria in the brain and liver we used the anti-RFP (MBL Co Ltd; 1:500). For the immunofluorescent staining, goat anti-rabbit Alexa Fluor 488 and goat anti-mouse cy-3 (Invitrogen, Carmarillo, CA, USA) were used as secondary Abs. Sections were covered with mounting medium with DAPI (Abcam). Briefly, tissue sections were fixed in 4% formaldehyde (10 min, room temperature), washed in Optimax (BioGenex Laboratories, San Ramon, CA, USA) and blocked in Cas Block (Invitrogen, Carmarillo, CA, USA) (10 min, room temperature). The sections were then incubated with the appropriate primary antibody (overnight at 40°C) and detected with the appropriate secondary antibody (1 hr, room temperature). Thioflavin and FJD histochemical stainings were conducted according to standard protocols. The sections were imaged by fluorescent microscopy (X20, Nikon-TL, or confocal microscope). Analyses done by ImageJ and Nis elements software [29, 31, 32].

Imaging analysis:

DsRed2-mitochondria or buffer only were IV injected to the tail of the AD-mice. About 2 hours later mice were IP anesthetized with 2 ml pental diluted with PBS (1:10). Brains and livers were collected into cold PBS, then put to imaging analysis for detection of the DsRed signal using the In Vitro Imaging System (IVIS Lumina LT Series III, Perkin Elmer). Excitation was at 556 nm, and Emission at 583 nm.

Proteomic studies

Sample preparation

Hippocampus samples were homogenized in 300 µl cold lysis buffer at 40ºC. Lysis buffer: 50 mM TrisHCl pH 8.8, 100 mM NaCl, 5 mM MgCl2, 0.5% (w/v) Nonident P-40 (NP-40),

1 mM EDTA, and 1:100 protease inhibitor cocktail. Homogenization was followed by 2 sonications. The samples were centrifuged at 15,000 x g for 15 min. The supernatant was transferred to a new tube – soluble fraction. The pellet was washed in 100 µl cold lysis buffer and centrifuged at 15,000 x g for 10 min. The supernatant was added to the soluble fraction. The pellet was resuspended in 350 µl of 100 mM Tris pH8, 10 mM DTT, 5% SDS and boiled for 5 min at 95ºC. Two sonications were performed followed by centrifugation at 14,000 g for 10 min. The supernatant was transferred to a new tube – aggregate fraction. The soluble and aggregate fractions were precipitated overnight in 80% acetone at -20ºC. The proteins were precipitated at 16,000 x g for 10 min at 4ºC. The protein pellets were washed in 2 ml cold 80% acetone and incubated for 15 min at -20ºC followed by centrifugation at 5,900 x g for 10 min at 4ºC. The wash step was repeated 3 times. The pellets were resuspended in 450 µl urea buffer for the soluble fraction and 150 µl for the aggregate fraction. Urea buffer: 9 M Urea and 400mM ammonium bicarbonate, 10mM DTT. Reduction was performed in 60ºC for 30 min, followed by modification with 35mM iodoacetamide in 100 mM ammonium bicarbonate (room temperature for 30 min) and digested in 1.5 M Urea, 25 mM ammonium bicarbonate with modified trypsin (Promega), overnight at 37ºC in a 1:50 enzyme-to-substrate ratio. An additional second trypsinization was performed for 4 hours. The tryptic peptides were acidified by addition of 1% formic acid and desalted using C18 tips (Homemade stage tips) dried and re-suspended in 0.1% Formic acid.

MS/MS analysis

The peptides were resolved by reverse-phase chromatography on 0.075 X 300-mm fused silica capillaries (J&W) packed with Reprosil reversed phase material (C18-AQ 3µm, Dr Maisch GmbH, Germany). The peptides were eluted with linear 120 minutes or 60 minutes gradient of 5 to 28% for the soluble or aggregate fractions,

respectively. Followed by 15 minutes gradient of 28 to 95% and 15 minutes at 95% acetonitrile with 0.1% formic acid in water at a flow rate of 0.15 μl/min. Mass spectrometry was performed by Q Exactive HF mass spectrometer (Thermo) in a positive mode using repetitively full MS scan followed by collision induces dissociation (HCD) of the 20 or 18 most dominant ions selected from the first MS scan for the soluble or aggregate fractions, respectively.

Proteomic data analysis

The mass spectrometry data was analyzed using the MaxQuant software 1.5.2.8 for peak picking and identification using the Andromeda search engine, searching against the Mus musculus proteome from the Uniprot database with mass tolerance of 6 ppm for the precursor masses and 20 ppm for the fragment ions. Oxidation on methionine, protein N-terminus acetylation, ubiquitination (K) and phosphorylation (STY) were accepted as variable modifications and carbamidomethyl on cysteine was accepted as static modifications. Minimal peptide length was set to seven amino acids and a maximum of two miscleavages was allowed. Peptide- and protein-level false discovery rates (FDRs) were filtered to 1% using the target-decoy strategy. Protein tables were filtered to eliminate the identifications from the reverse database, and common contaminants and single peptide identifications. The data was quantified by label free analysis using the same software, based on extracted ion currents (XICs) of peptides enabling quantitation from each LC/MS run for each peptide identified in any of experiments. The analysis was done after recalibration of the retention times.

Statistical analysis of the identification and quantization results was done using Perseus 1.6.7.0 software (Mathias Mann's group). Hierarchical clustering of the differentially expressed proteins was performed to determine the differences among disease groups. Identification of the amino-acid motifs of the differential

phospho-peptides between the AD and AD-Treated groups in the brain samples done with the Phosphosite plus software.

Annotation by KEGG, GOTERM, Gorilla, STRING databases

To address to which pathways and terms do the proteins altered in response to treatment - belong, the full list of proteins were analyzed by the Gene Ontology enRIchment anaLysis and visuaLizAtion tool (GORILLA) "biological processes", "cellular component" and "molecular functions" algorithm, which allows testing of enriched components and processes while taking into consideration a ranking of protein list. Proteins detected by MS were ranked according to their intensity, which correlates with their abundance in the sample. We also analyzed the significantly (or having a trend) altered genes by enrichment analysis in the KEGG- Kyoto Encyclopedia of Genes and Genomes (KEGG) / GOTERM pathway analysis tool. To learn about both direct (physical) and indirect (functional) protein-protein interactions we used STRING-DB (version 11.5).

Metabolomic studies (Targeted Metabolomics)

we used the AbsoluteIDQ® p180 kit (Biocrates Life Sciences AG, Innsbruck, Austria), targeting 40 acylcarnitines, 42 amino acids/biogenic amines, 90 glycerophospholipids, 15 sphingolipids, and sum of hexoses, following the manufacturer's instructions. All reagents, internal standards (IS), calibration standards, quality controls (QCs), and the test mixes required for the AbsoluteIDQ® p180 analysis were included in the kit.

Sample preparation

Briefly, 10 μL of calibration standards, QCs, and treated samples were added onto the respective well filter of the 96-well Biocrates sample preparation plate containing a mix of internal standards. After drying the samples under nitrogen, 50 μL of 5% phenylisothiocyanate solution were added to each well for derivatization. After incubation for 25 min and subsequent evaporation to dryness

under nitrogen, 300 µL of 5 mM ammonium acetate in methanol were added for metabolite extraction, stirred for 30 min and centrifuged. The extracts were diluted with defined volumes of methanol/water according to the manufacturer's protocol.

The liver extracts and the sera were analyzed using the LC-MS/MS system. The LC-MS/MS was comprised of a Nexera UHPLC system (Shimadzu, Kyoto, Japan) coupled to a Triple Quad™ 5500 mass spectrometer (Sciex, Framingham, MA, USA) in electrospray ionization (ESI) mode. Amino acids and biogenic amines were analyzed via LC-MS in positive mode. 5 µL of the sample extract were injected onto a Biocrates AbsoluteIDQ® p180 kit UHPLC column, 2.1 × 50 mm, protected by a VanGuard® pre-column (Waters, Milford, MA, USA) at 50 °C using a 5.8 min solvent gradient employing 0.2% formic acid in water (solvent A) and 0.2% formic acid in acetonitrile (solvent B). Twenty µL of the sample extracts were used in the flow injection analysis (FIA) in positive mode to capture acylcarnitines, glycerophospholipids, sphingolipids and hexoses. All FIA injections were carried out using the Biocrates FIA Solvent. Additional LC and MS settings for LC-MS and FIA mode are described in Table 1. All metabolites were identified and quantified using isotopically-labeled internal standards and multiple reaction monitoring (MRM).

Data Processing and Statistical Analysis

LC-MS raw data were quantified using Analyst 1.6.3 software (Sciex) and exported to the Biocrates MetIDQ™ software. FIA raw data of the AbsoluteIDQ® assay were exported and quantified using MetIDQ™ software. Quality control samples-based data normalization was performed to minimize the variation of analyses. Initial data cleaning was performed by excluding metabolites with >20% missing values or values below the limit of detection (LOD) in all experimental groups. Thus, all metabolites with >80% of the concentration values above the LOD in at least one

of the four experimental groups were included for statistical analysis. Remaining missing values were replaced by 1/5 of the minimum positive value of each variable. Data were log-transformed to assume normal distribution before comprehensive downstream analysis using the web-based tool MetaboAnalyst 5.0 (https://www.metaboanalyst.ca) [35]. Partial least square discriminant analtysis (PLS-DA) was performed whenever necessary to determine the metabolic signature contributing to group separation. PLS-DA decreases intergroup variability and improves separation. However, PLS-DA is prone to data overfitting. Thus, the quality of the model was assessed by cross-validation (calculation of Q2, R2, and accuracy values) and the overfitting tendency of the model was validated using permutation testing. The PLS-DA Variable Importance in Projection (VIP-score) was calculated and metabolites with a VIP score > 1 were considered important for group separation. Thus, for Metabolomic Pathway Analysis (MetPA) we used the KEGG pathway libraries as references. Pearson coefficient-based correlation analysis was conducted either against a selected metabolite, or against a pattern such as the transition from one group (e.g., wild type) to another (e.g., transgenic). Correlation analyses and heatmaps were also generated by MetaboAnalyst 5.0.
